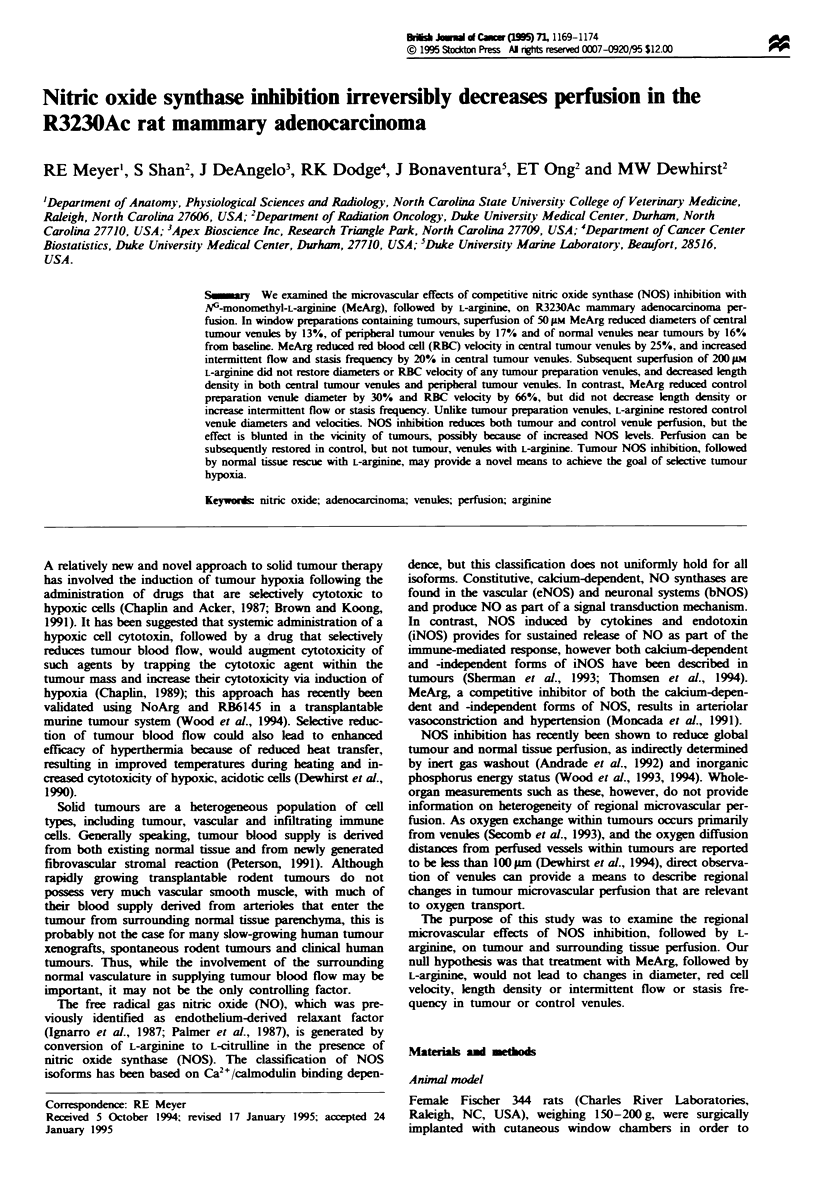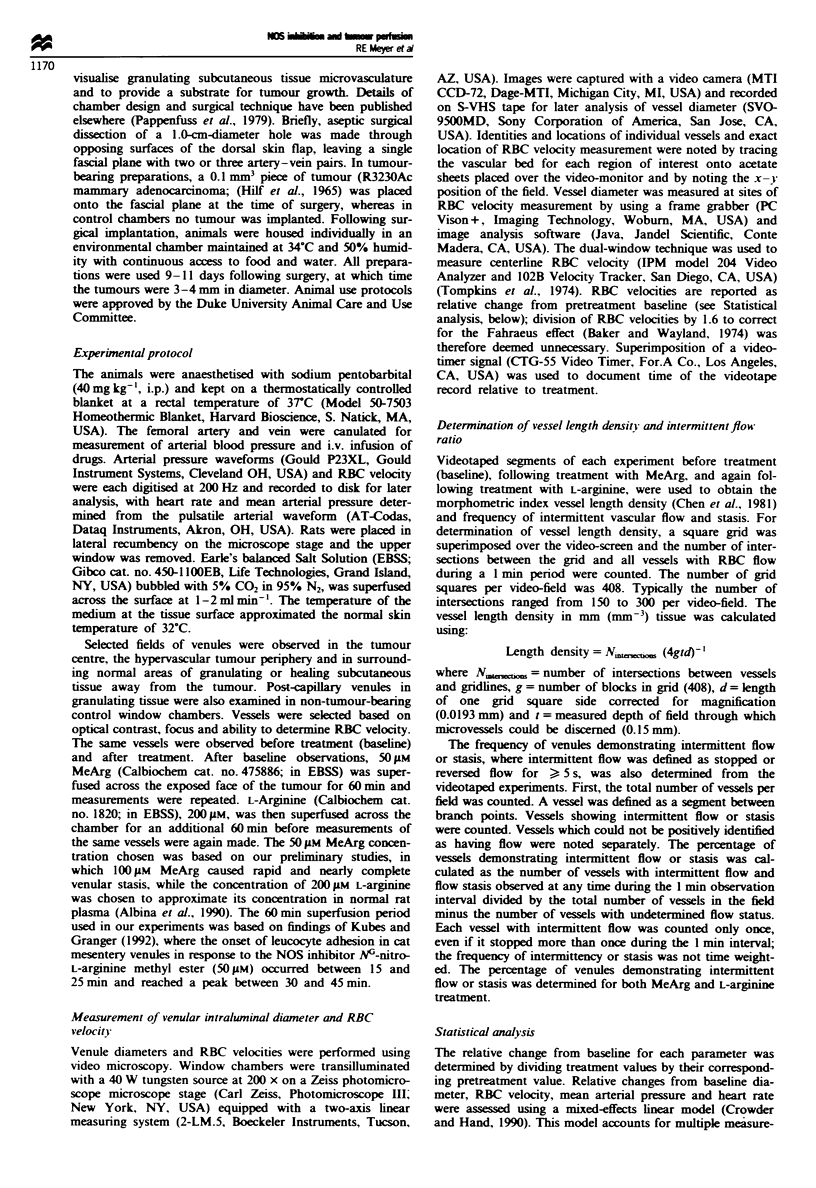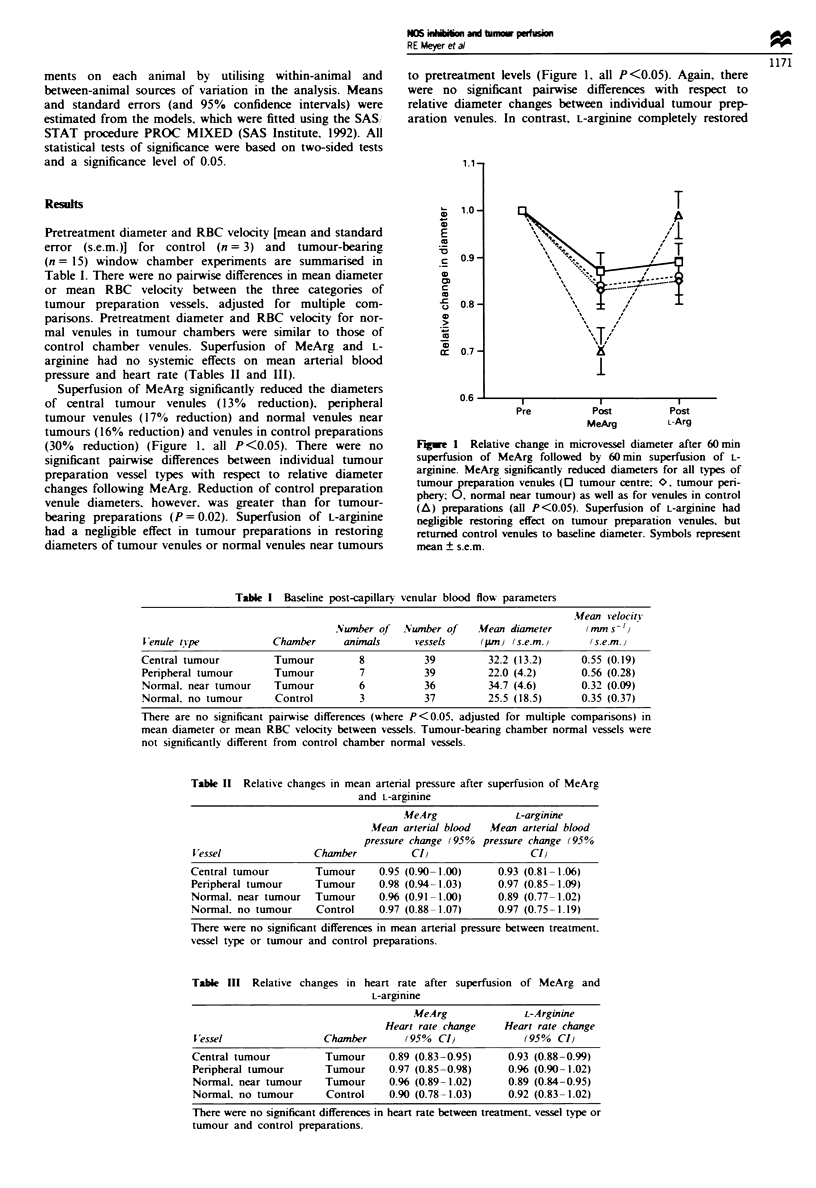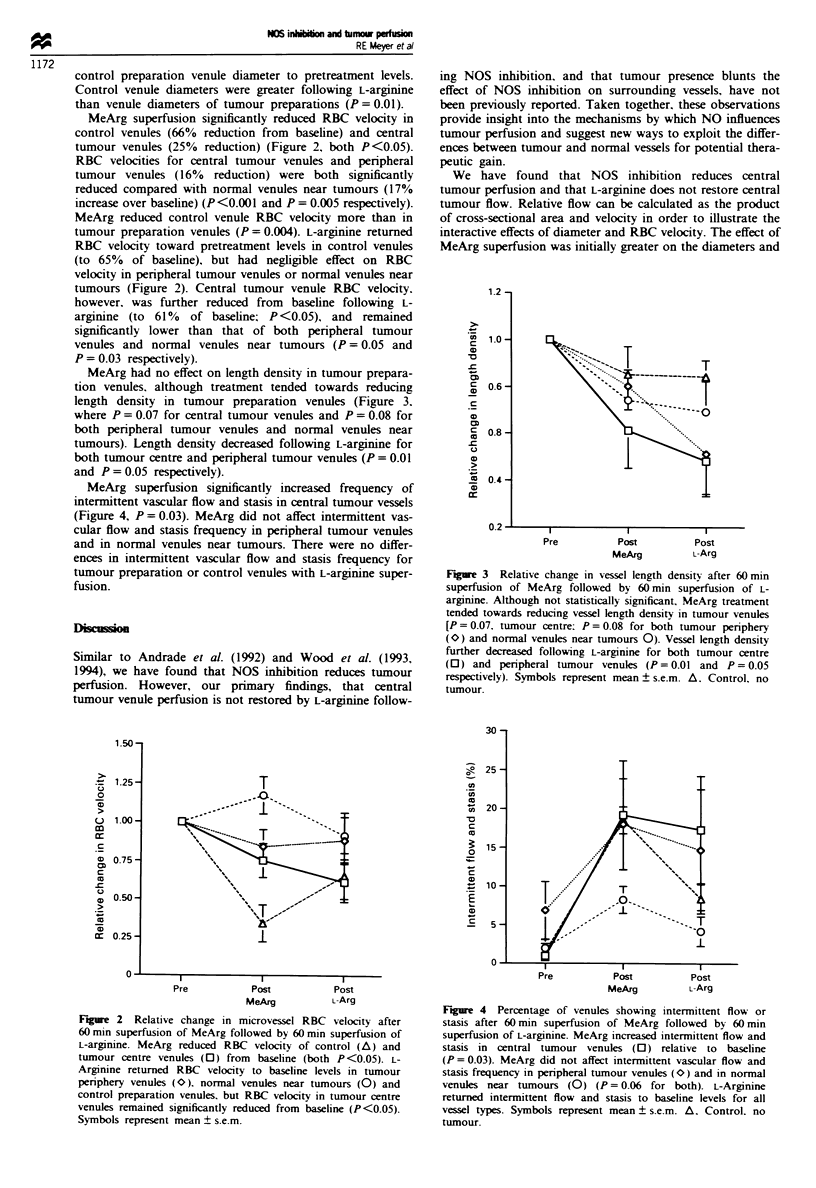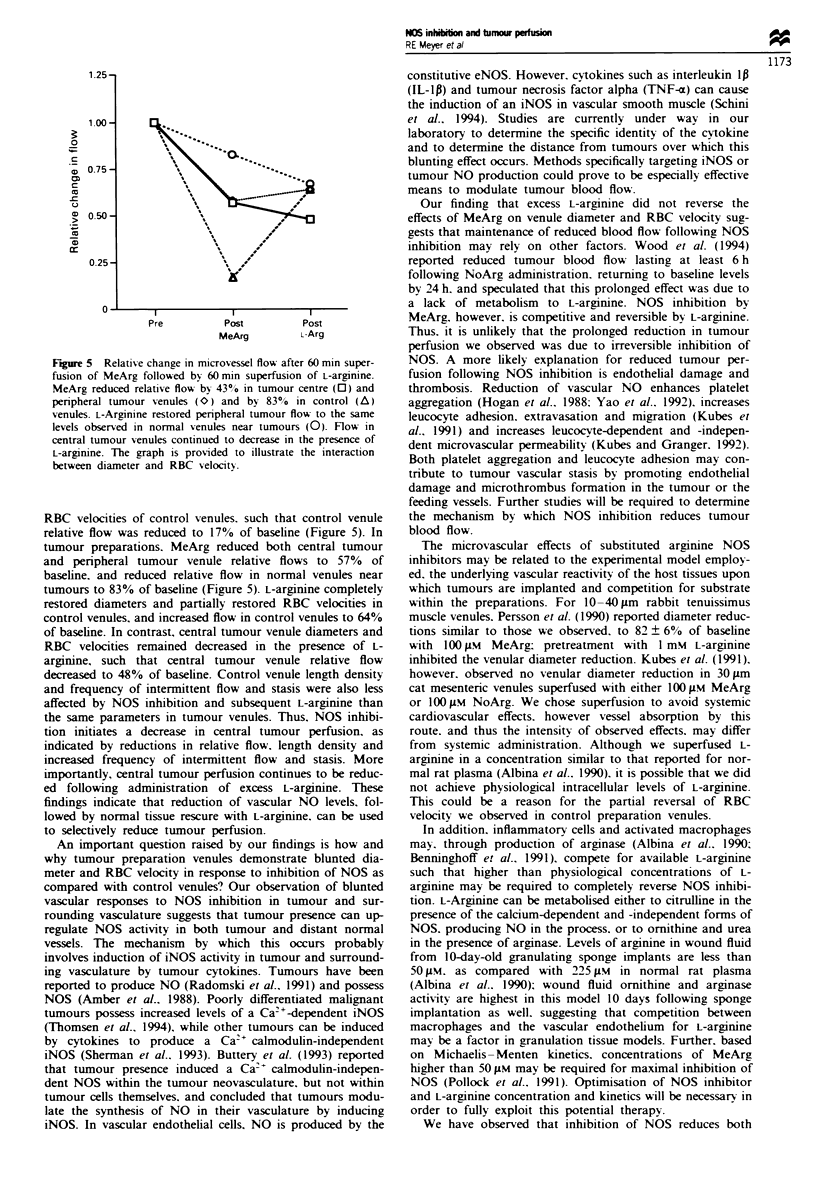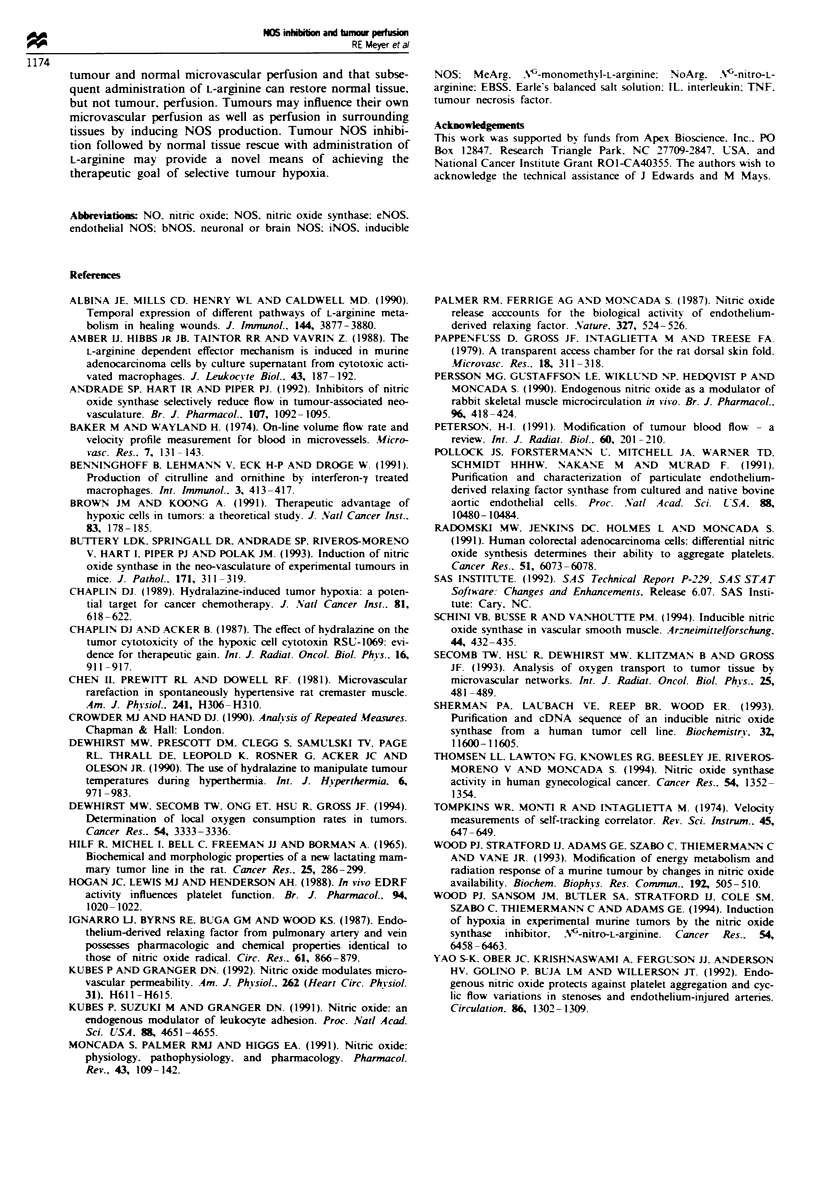# Nitric oxide synthase inhibition irreversibly decreases perfusion in the R3230Ac rat mammary adenocarcinoma.

**DOI:** 10.1038/bjc.1995.228

**Published:** 1995-06

**Authors:** R. E. Meyer, S. Shan, J. DeAngelo, R. K. Dodge, J. Bonaventura, E. T. Ong, M. W. Dewhirst

**Affiliations:** Department of Anatomy, North Carolina State University College of Veterinary Medicine, Raleigh 27606, USA.

## Abstract

We examined the microvascular effects of competitive nitric oxide synthase (NOS) inhibition with NG-monomethyl-L-arginine (MeArg), followed by L-arginine, on R3230Ac mammary adenocarcinoma perfusion. In window preparations containing tumours, superfusion of 50 microM MeArg reduced diameters of central tumour venules by 13%, of peripheral tumour venules by 17% and of normal venules near tumours by 16% from baseline. MeArg reduced red blood cell (RBC) velocity in central tumour venules by 25%, and increased intermittent flow and stasis frequency by 20% in central tumour venules. Subsequent superfusion of 200 microM L-arginine did not restore diameters or RBC velocity of any tumour preparation venules, and decreased length density in both central tumour venules and peripheral tumour venules. In contrast, MeArg reduced control preparation venule diameter by 30% and RBC velocity by 66%, but did not decrease length density or increase intermittent flow or stasis frequency. Unlike tumour preparation venules, L-arginine restored control venule diameters and velocities. NOS inhibition reduces both tumour and control venule perfusion, but the effect is blunted in the vicinity of tumours, possibly because of increased NOS levels. Perfusion can be subsequently restored in control, but not tumour, venules with L-arginine. Tumour NOS inhibition, followed by normal tissue rescue with L-arginine, may provide a novel means to achieve the goal of selective tumour hypoxia.


© 1995 Stockton Press All rights reserved 0007-0920/95 $12.00

### Nitric oxide synthase inhibition irreversibly decreases perfusion in the R3230Ac rat mammary adenocarcinoma

RE Meyer[1], S Shan[2], J DeAngelo[3], RK Dodge[4], J Bonaventura[5], ET Ong[2] and MW Dewhirst[2]

[1]*Department of Anatomy, Physiological Sciences and Radiology, North Carolina State University College of Veterinary Medicine, Raleigh, North Carolina 27606, USA;* [2]*Department of Radiation Oncology, Duke University Medical Center, Durham, North Carolina 27710, USA;* [3]*Apex Bioscience Inc, Research Triangle Park, North Carolina 27709, USA;* [4]*Department of Cancer Center Biostatistics, Duke University Medical Center, Durham, 27710, USA;* [5]*Duke University Marine Laboratory, Beaufort, 28516, USA.*

**Summary** We examined the microvascular effects of competitive nitric oxide synthase (NOS) inhibition with $N^G$-monomethyl-L-arginine (MeArg), followed by L-arginine, on R3230Ac mammary adenocarcinoma perfusion. In window preparations containing tumours, superfusion of 50 μM MeArg reduced diameters of central tumour venules by 13%, of peripheral tumour venules by 17% and of normal venules near tumours by 16% from baseline. MeArg reduced red blood cell (RBC) velocity in central tumour venules by 25%, and increased intermittent flow and stasis frequency by 20% in central tumour venules. Subsequent superfusion of 200 μM L-arginine did not restore diameters or RBC velocity of any tumour preparation venules, and decreased length density in both central tumour venules and peripheral tumour venules. In contrast, MeArg reduced control preparation venule diameter by 30% and RBC velocity by 66%, but did not decrease length density or increase intermittent flow or stasis frequency. Unlike tumour preparation venules, L-arginine restored control venule diameters and velocities. NOS inhibition reduces both tumour and control venule perfusion, but the effect is blunted in the vicinity of tumours, possibly because of increased NOS levels. Perfusion can be subsequently restored in control, but not tumour, venules with L-arginine. Tumour NOS inhibition, followed by normal tissue rescue with L-arginine, may provide a novel means to achieve the goal of selective tumour hypoxia.

**Keywords:** nitric oxide; adenocarcinoma; venules; perfusion; arginine

A relatively new and novel approach to solid tumour therapy has involved the induction of tumour hypoxia following the administration of drugs that are selectively cytotoxic to hypoxic cells (Chaplin and Acker, 1987; Brown and Koong, 1991). It has been suggested that systemic administration of a hypoxic cell cytotoxin, followed by a drug that selectively reduces tumour blood flow, would augment cytotoxicity of such agents by trapping the cytotoxic agent within the tumour mass and increase their cytotoxicity via induction of hypoxia (Chaplin, 1989); this approach has recently been validated using NoArg and RB6145 in a transplantable murine tumour system (Wood *et al.*, 1994). Selective reduction of tumour blood flow could also lead to enhanced efficacy of hyperthermia because of reduced heat transfer, resulting in improved temperatures during heating and increased cytotoxicity of hypoxic, acidotic cells (Dewhirst *et al.*, 1990).

Solid tumours are a heterogeneous population of cell types, including tumour, vascular and infiltrating immune cells. Generally speaking, tumour blood supply is derived from both existing normal tissue and from newly generated fibrovascular stromal reaction (Peterson, 1991). Although rapidly growing transplantable rodent tumours do not possess very much vascular smooth muscle, with much of their blood supply derived from arterioles that enter the tumour from surrounding normal tissue parenchyma, this is probably not the case for many slow-growing human tumour xenografts, spontaneous rodent tumours and clinical human tumours. Thus, while the involvement of the surrounding normal vasculature in supplying tumour blood flow may be important, it may not be the only controlling factor.

The free radical gas nitric oxide (NO), which was previously identified as endothelium-derived relaxant factor (Ignarro *et al.*, 1987; Palmer *et al.*, 1987), is generated by conversion of L-arginine to L-citrulline in the presence of nitric oxide synthase (NOS). The classification of NOS isoforms has been based on $Ca^{2+}$/calmodulin binding dependence, but this classification does not uniformly hold for all isoforms. Constitutive, calcium-dependent, NO synthases are found in the vascular (eNOS) and neuronal systems (bNOS) and produce NO as part of a signal transduction mechanism. In contrast, NOS induced by cytokines and endotoxin (iNOS) provides for sustained release of NO as part of the immune-mediated response, however both calcium-dependent and -independent forms of iNOS have been described in tumours (Sherman *et al.*, 1993; Thomsen *et al.*, 1994). MeArg, a competitive inhibitor of both the calcium-dependent and -independent forms of NOS, results in arteriolar vasoconstriction and hypertension (Moncada *et al.*, 1991).

NOS inhibition has recently been shown to reduce global tumour and normal tissue perfusion, as indirectly determined by inert gas washout (Andrade *et al.*, 1992) and inorganic phosphorus energy status (Wood *et al.*, 1993, 1994). Whole-organ measurements such as these, however, do not provide information on heterogeneity of regional microvascular perfusion. As oxygen exchange within tumours occurs primarily from venules (Secomb *et al.*, 1993), and the oxygen diffusion distances from perfused vessels within tumours are reported to be less than 100 μm (Dewhirst *et al.*, 1994), direct observation of venules can provide a means to describe regional changes in tumour microvascular perfusion that are relevant to oxygen transport.

The purpose of this study was to examine the regional microvascular effects of NOS inhibition, followed by L-arginine, on tumour and surrounding tissue perfusion. Our null hypothesis was that treatment with MeArg, followed by L-arginine, would not lead to changes in diameter, red cell velocity, length density or intermittent flow or stasis frequency in tumour or control venules.

### Materials and methods

### *Animal model*

Female Fischer 344 rats (Charles River Laboratories, Raleigh, NC, USA), weighing 150-200 g, were surgically implanted with cutaneous window chambers in order to

Correspondence: RE Meyer
Received 5 October 1994; revised 17 January 1995; accepted 24 January 1995

visualise granulating subcutaneous tissue microvasculature and to provide a substrate for tumour growth. Details of chamber design and surgical technique have been published elsewhere (Pappenfuss *et al*., 1979). Briefly, aseptic surgical dissection of a 1.0-cm-diameter hole was made through opposing surfaces of the dorsal skin flap, leaving a single fascial plane with two or three artery–vein pairs. In tumour-bearing preparations, a 0.1 $mm^3$ piece of tumour (R3230Ac mammary adenocarcinoma; (Hilf *et al*., 1965) was placed onto the fascial plane at the time of surgery, whereas in control chambers no tumour was implanted. Following surgical implantation, animals were housed individually in an environmental chamber maintained at 34°C and 50% humidity with continuous access to food and water. All preparations were used 9–11 days following surgery, at which time the tumours were 3–4 mm in diameter. Animal use protocols were approved by the Duke University Animal Care and Use Committee.

*Experimental protocol*

The animals were anaesthetised with sodium pentobarbital (40 mg $kg^{-1}$, i.p.) and kept on a thermostatically controlled blanket at a rectal temperature of 37°C (Model 50-7503 Homeothermic Blanket, Harvard Bioscience, S. Natick, MA, USA). The femoral artery and vein were canulated for measurement of arterial blood pressure and i.v. infusion of drugs. Arterial pressure waveforms (Gould P23XL, Gould Instrument Systems, Cleveland OH, USA) and RBC velocity were each digitised at 200 Hz and recorded to disk for later analysis, with heart rate and mean arterial pressure determined from the pulsatile arterial waveform (AT-Codas, Dataq Instruments, Akron, OH, USA). Rats were placed in lateral recumbency on the microscope stage and the upper window was removed. Earle's balanced Salt Solution (EBSS; Gibco cat. no. 450-1100EB, Life Technologies, Grand Island, NY, USA) bubbled with 5% $CO_2$ in 95% $N_2$, was superfused across the surface at 1–2 ml $min^{-1}$. The temperature of the medium at the tissue surface approximated the normal skin temperature of 32°C.

Selected fields of venules were observed in the tumour centre, the hypervascular tumour periphery and in surrounding normal areas of granulating or healing subcutaneous tissue away from the tumour. Post-capillary venules in granulating tissue were also examined in non-tumour-bearing control window chambers. Vessels were selected based on optical contrast, focus and ability to determine RBC velocity. The same vessels were observed before treatment (baseline) and after treatment. After baseline observations, 50 μM MeArg (Calbiochem cat. no. 475886; in EBSS) was superfused across the exposed face of the tumour for 60 min and measurements were repeated. L-Arginine (Calbiochem cat. no. 1820; in EBSS), 200 μM, was then superfused across the chamber for an additional 60 min before measurements of the same vessels were again made. The 50 μM MeArg concentration chosen was based on our preliminary studies, in which 100 μM MeArg caused rapid and nearly complete venular stasis, while the concentration of 200 μM L-arginine was chosen to approximate its concentration in normal rat plasma (Albina *et al*., 1990). The 60 min superfusion period used in our experiments was based on findings of Kubes and Granger (1992), where the onset of leucocyte adhesion in cat mesentery venules in response to the NOS inhibitor $N^G$-nitro-L-arginine methyl ester (50 μM) occurred between 15 and 25 min and reached a peak between 30 and 45 min.

*Measurement of venular intraluminal diameter and RBC velocity*

Venule diameters and RBC velocities were performed using video microscopy. Window chambers were transilluminated with a 40 W tungsten source at 200 × on a Zeiss photomicroscope microscope stage (Carl Zeiss, Photomicroscope III; New York, NY, USA) equipped with a two-axis linear measuring system (2-LM.5, Boeckeler Instruments, Tucson, AZ, USA). Images were captured with a video camera (MTI CCD-72, Dage-MTI, Michigan City, MI, USA) and recorded on S-VHS tape for later analysis of vessel diameter (SVO-9500MD, Sony Corporation of America, San Jose, CA, USA). Identities and locations of individual vessels and exact location of RBC velocity measurement were noted by tracing the vascular bed for each region of interest onto acetate sheets placed over the video-monitor and by noting the $x$–$y$ position of the field. Vessel diameter was measured at sites of RBC velocity measurement by using a frame grabber (PC Vison+, Imaging Technology, Woburn, MA, USA) and image analysis software (Java, Jandel Scientific, Conte Madera, CA, USA). The dual-window technique was used to measure centerline RBC velocity (IPM model 204 Video Analyzer and 102B Velocity Tracker, San Diego, CA, USA) (Tompkins *et al*., 1974). RBC velocities are reported as relative change from pretreatment baseline (see Statistical analysis, below); division of RBC velocities by 1.6 to correct for the Fahraeus effect (Baker and Wayland, 1974) was therefore deemed unnecessary. Superimposition of a video-timer signal (CTG-55 Video Timer, For.A Co., Los Angeles, CA, USA) was used to document time of the videotape record relative to treatment.

*Determination of vessel length density and intermittent flow ratio*

Videotaped segments of each experiment before treatment (baseline), following treatment with MeArg, and again following treatment with L-arginine, were used to obtain the morphometric index vessel length density (Chen *et al*., 1981) and frequency of intermittent vascular flow and stasis. For determination of vessel length density, a square grid was superimposed over the video-screen and the number of intersections between the grid and all vessels with RBC flow during a 1 min period were counted. The number of grid squares per video-field was 408. Typically the number of intersections ranged from 150 to 300 per video-field. The vessel length density in mm ($mm^{-3}$) tissue was calculated using:

$$\text{Length density} = N_{\text{intersections}}\,(4gtd)^{-1}$$

where $N_{\text{intersections}}$ = number of intersections between vessels and gridlines, $g$ = number of blocks in grid (408), $d$ = length of one grid square side corrected for magnification (0.0193 mm) and $t$ = measured depth of field through which microvessels could be discerned (0.15 mm).

The frequency of venules demonstrating intermittent flow or stasis, where intermittent flow was defined as stopped or reversed flow for ⩾5 s, was also determined from the videotaped experiments. First, the total number of vessels per field was counted. A vessel was defined as a segment between branch points. Vessels showing intermittent flow or stasis were counted. Vessels which could not be positively identified as having flow were noted separately. The percentage of vessels demonstrating intermittent flow or stasis was calculated as the number of vessels with intermittent flow and flow stasis observed at any time during the 1 min observation interval divided by the total number of vessels in the field minus the number of vessels with undetermined flow status. Each vessel with intermittent flow was counted only once, even if it stopped more than once during the 1 min interval; the frequency of intermittency or stasis was not time weighted. The percentage of venules demonstrating intermittent flow or stasis was determined for both MeArg and L-arginine treatment.

*Statistical analysis*

The relative change from baseline for each parameter was determined by dividing treatment values by their corresponding pretreatment value. Relative changes from baseline diameter, RBC velocity, mean arterial pressure and heart rate were assessed using a mixed-effects linear model (Crowder and Hand, 1990). This model accounts for multiple measurements on each animal by utilising within-animal and between-animal sources of variation in the analysis. Means and standard errors (and 95% confidence intervals) were estimated from the models, which were fitted using the SAS/STAT procedure PROC MIXED (SAS Institute, 1992). All statistical tests of significance were based on two-sided tests and a significance level of 0.05.

### Results

Pretreatment diameter and RBC velocity [mean and standard error (s.e.m.)] for control ($n = 3$) and tumour-bearing ($n = 15$) window chamber experiments are summarised in Table I. There were no pairwise differences in mean diameter or mean RBC velocity between the three categories of tumour preparation vessels, adjusted for multiple comparisons. Pretreatment diameter and RBC velocity for normal venules in tumour chambers were similar to those of control chamber venules. Superfusion of MeArg and L-arginine had no systemic effects on mean arterial blood pressure and heart rate (Tables II and III).

Superfusion of MeArg significantly reduced the diameters of central tumour venules (13% reduction), peripheral tumour venules (17% reduction) and normal venules near tumours (16% reduction) and venules in control preparations (30% reduction) (Figure 1, all $P<0.05$). There were no significant pairwise differences between individual tumour preparation vessel types with respect to relative diameter changes following MeArg. Reduction of control preparation venule diameters, however, was greater than for tumour-bearing preparations ($P = 0.02$). Superfusion of L-arginine had a negligible effect in tumour preparations in restoring diameters of tumour venules or normal venules near tumours to pretreatment levels (Figure 1, all $P<0.05$). Again, there were no significant pairwise differences with respect to relative diameter changes between individual tumour preparation venules. In contrast, L-arginine completely restored

**Figure 1** Relative change in microvessel diameter after 60 min superfusion of MeArg followed by 60 min superfusion of L-arginine. MeArg significantly reduced diameters for all types of tumour preparation venules (□ tumour centre; ◇, tumour periphery; O, normal near tumour) as well as for venules in control (Δ) preparations (all $P<0.05$). Superfusion of L-arginine had negligible restoring effect on tumour preparation venules, but returned control venules to baseline diameter. Symbols represent mean ± s.e.m.

**Table I** Baseline post-capillary venular blood flow parameters

| Venule type | Chamber | Number of animals | Number of vessels | Mean diameter (μm) (s.e.m.) | Mean velocity (mm $s^{-1}$) (s.e.m.) |
|---|---|---|---|---|---|
| Central tumour | Tumour | 8 | 39 | 32.2 (13.2) | 0.55 (0.19) |
| Peripheral tumour | Tumour | 7 | 39 | 22.0 (4.2) | 0.56 (0.28) |
| Normal, near tumour | Tumour | 6 | 36 | 34.7 (4.6) | 0.32 (0.09) |
| Normal, no tumour | Control | 3 | 37 | 25.5 (18.5) | 0.35 (0.37) |

There are no significant pairwise differences (where $P<0.05$, adjusted for multiple comparisons) in mean diameter or mean RBC velocity between vessels. Tumour-bearing chamber normal vessels were not significantly different from control chamber normal vessels.

**Table II** Relative changes in mean arterial pressure after superfusion of MeArg and L-arginine

| Vessel | Chamber | MeArg Mean arterial blood pressure change (95% CI) | L-arginine Mean arterial blood pressure change (95% CI) |
|---|---|---|---|
| Central tumour | Tumour | 0.95 (0.90-1.00) | 0.93 (0.81-1.06) |
| Peripheral tumour | Tumour | 0.98 (0.94-1.03) | 0.97 (0.85-1.09) |
| Normal, near tumour | Tumour | 0.96 (0.91-1.00) | 0.89 (0.77-1.02) |
| Normal, no tumour | Control | 0.97 (0.88-1.07) | 0.97 (0.75-1.19) |

There were no significant differences in mean arterial pressure between treatment, vessel type or tumour and control preparations.

**Table III** Relative changes in heart rate after superfusion of MeArg and L-arginine

| Vessel | Chamber | MeArg Heart rate change (95% CI) | L-Arginine Heart rate change (95% CI) |
|---|---|---|---|
| Central tumour | Tumour | 0.89 (0.83-0.95) | 0.93 (0.88-0.99) |
| Peripheral tumour | Tumour | 0.97 (0.85-0.98) | 0.96 (0.90-1.02) |
| Normal, near tumour | Tumour | 0.96 (0.89-1.02) | 0.89 (0.84-0.95) |
| Normal, no tumour | Control | 0.90 (0.78-1.03) | 0.92 (0.83-1.02) |

There were no significant differences in heart rate between treatment, vessel type or tumour and control preparations.

control preparation venule diameter to pretreatment levels. Control venule diameters were greater following L-arginine than venule diameters of tumour preparations ($P = 0.01$).

MeArg superfusion significantly reduced RBC velocity in control venules (66% reduction from baseline) and central tumour venules (25% reduction) (Figure 2, both $P < 0.05$). RBC velocities for central tumour venules and peripheral tumour venules (16% reduction) were both significantly reduced compared with normal venules near tumours (17% increase over baseline) ($P < 0.001$ and $P = 0.005$ respectively). MeArg reduced control venule RBC velocity more than in tumour preparation venules ($P = 0.004$). L-arginine returned RBC velocity toward pretreatment levels in control venules (to 65% of baseline), but had negligible effect on RBC velocity in peripheral tumour venules or normal venules near tumours (Figure 2). Central tumour venule RBC velocity, however, was further reduced from baseline following L-arginine (to 61% of baseline; $P < 0.05$), and remained significantly lower than that of both peripheral tumour venules and normal venules near tumours ($P = 0.05$ and $P = 0.03$ respectively).

MeArg had no effect on length density in tumour preparation venules, although treatment tended towards reducing length density in tumour preparation venules (Figure 3, where $P = 0.07$ for central tumour venules and $P = 0.08$ for both peripheral tumour venules and normal venules near tumours). Length density decreased following L-arginine for both tumour centre and peripheral tumour venules ($P = 0.01$ and $P = 0.05$ respectively).

MeArg superfusion significantly increased frequency of intermittent vascular flow and stasis in central tumour vessels (Figure 4, $P = 0.03$). MeArg did not affect intermittent vascular flow and stasis frequency in peripheral tumour venules and in normal venules near tumours. There were no differences in intermittent vascular flow and stasis frequency for tumour preparation or control venules with L-arginine superfusion.

### Discussion

Similar to Andrade et al. (1992) and Wood et al. (1993, 1994), we have found that NOS inhibition reduces tumour perfusion. However, our primary findings, that central tumour venule perfusion is not restored by L-arginine following NOS inhibition, and that tumour presence blunts the effect of NOS inhibition on surrounding vessels, have not been previously reported. Taken together, these observations provide insight into the mechanisms by which NO influences tumour perfusion and suggest new ways to exploit the differences between tumour and normal vessels for potential therapeutic gain.

We have found that NOS inhibition reduces central tumour perfusion and that L-arginine does not restore central tumour flow. Relative flow can be calculated as the product of cross-sectional area and velocity in order to illustrate the interactive effects of diameter and RBC velocity. The effect of MeArg superfusion was initially greater on the diameters and

**Figure 3** Relative change in vessel length density after 60 min superfusion of MeArg followed by 60 min superfusion of L-arginine. Although not statistically significant, MeArg treatment tended towards reducing vessel length density in tumour venules [$P = 0.07$, tumour centre; $P = 0.08$ for both tumour periphery (◇) and normal venules near tumours ○). Vessel length density further decreased following L-arginine for both tumour centre (□) and peripheral tumour venules ($P = 0.01$ and $P = 0.05$ respectively). Symbols represent mean ± s.e.m. △, Control, no tumour.

**Figure 2** Relative change in microvessel RBC velocity after 60 min superfusion of MeArg followed by 60 min superfusion of L-arginine. MeArg reduced RBC velocity of control (△) and tumour centre venules (□) from baseline (both $P < 0.05$). L-Arginine returned RBC velocity to baseline levels in tumour periphery venules (◇), normal venules near tumours (○) and control preparation venules, but RBC velocity in tumour centre venules remained significantly reduced from baseline ($P < 0.05$). Symbols represent mean ± s.e.m.

**Figure 4** Percentage of venules showing intermittent flow or stasis after 60 min superfusion of MeArg followed by 60 min superfusion of L-arginine. MeArg increased intermittent flow and stasis in central tumour venules (□) relative to baseline ($P = 0.03$). MeArg did not affect intermittent vascular flow and stasis frequency in peripheral tumour venules (◇) and in normal venules near tumours (○) ($P = 0.06$ for both). L-Arginine returned intermittent flow and stasis to baseline levels for all vessel types. Symbols represent mean ± s.e.m. △, Control, no tumour.

**Figure 5** Relative change in microvessel flow after 60 min superfusion of MeArg followed by 60 min superfusion of L-arginine. MeArg reduced relative flow by 43% in tumour centre (□) and peripheral tumour venules (◇) and by 83% in control (△) venules. L-Arginine restored peripheral tumour flow to the same levels observed in normal venules near tumours (○). Flow in central tumour venules continued to decrease in the presence of L-arginine. The graph is provided to illustrate the interaction between diameter and RBC velocity.

RBC velocities of control venules, such that control venule relative flow was reduced to 17% of baseline (Figure 5). In tumour preparations, MeArg reduced both central tumour and peripheral tumour venule relative flows to 57% of baseline, and reduced relative flow in normal venules near tumours to 83% of baseline (Figure 5). L-arginine completely restored diameters and partially restored RBC velocities in control venules, and increased flow in control venules to 64% of baseline. In contrast, central tumour venule diameters and RBC velocities remained decreased in the presence of L-arginine, such that central tumour venule relative flow decreased to 48% of baseline. Control venule length density and frequency of intermittent flow and stasis were also less affected by NOS inhibition and subsequent L-arginine than the same parameters in tumour venules. Thus, NOS inhibition initiates a decrease in central tumour perfusion, as indicated by reductions in relative flow, length density and increased frequency of intermittent flow and stasis. More importantly, central tumour perfusion continues to be reduced following administration of excess L-arginine. These findings indicate that reduction of vascular NO levels, followed by normal tissue rescure with L-arginine, can be used to selectively reduce tumour perfusion.

An important question raised by our findings is how and why tumour preparation venules demonstrate blunted diameter and RBC velocity in response to inhibition of NOS as compared with control venules? Our observation of blunted vascular responses to NOS inhibition in tumour and surrounding vasculature suggests that tumour presence can upregulate NOS activity in both tumour and distant normal vessels. The mechanism by which this occurs probably involves induction of iNOS activity in tumour and surrounding vasculature by tumour cytokines. Tumours have been reported to produce NO (Radomski *et al.*, 1991) and possess NOS (Amber *et al.*, 1988). Poorly differentiated malignant tumours possess increased levels of a $Ca^{2+}$-dependent iNOS (Thomsen *et al.*, 1994), while other tumours can be induced by cytokines to produce a $Ca^{2+}$ calmodulin-independent iNOS (Sherman *et al.*, 1993). Buttery *et al.* (1993) reported that tumour presence induced a $Ca^{2+}$ calmodulin-independent NOS within the tumour neovasculature, but not within tumour cells themselves, and concluded that tumours modulate the synthesis of NO in their vasculature by inducing iNOS. In vascular endothelial cells, NO is produced by the constitutive eNOS. However, cytokines such as interleukin 1β (IL-1β) and tumour necrosis factor alpha (TNF-α) can cause the induction of an iNOS in vascular smooth muscle (Schini *et al.*, 1994). Studies are currently under way in our laboratory to determine the specific identity of the cytokine and to determine the distance from tumours over which this blunting effect occurs. Methods specifically targeting iNOS or tumour NO production could prove to be especially effective means to modulate tumour blood flow.

Our finding that excess L-arginine did not reverse the effects of MeArg on venule diameter and RBC velocity suggests that maintenance of reduced blood flow following NOS inhibition may rely on other factors. Wood *et al.* (1994) reported reduced tumour blood flow lasting at least 6 h following NoArg administration, returning to baseline levels by 24 h, and speculated that this prolonged effect was due to a lack of metabolism to L-arginine. NOS inhibition by MeArg, however, is competitive and reversible by L-arginine. Thus, it is unlikely that the prolonged reduction in tumour perfusion we observed was due to irreversible inhibition of NOS. A more likely explanation for reduced tumour perfusion following NOS inhibition is endothelial damage and thrombosis. Reduction of vascular NO enhances platelet aggregation (Hogan *et al.*, 1988; Yao *et al.*, 1992), increases leucocyte adhesion, extravasation and migration (Kubes *et al.*, 1991) and increases leucocyte-dependent and -independent microvascular permeability (Kubes and Granger, 1992). Both platelet aggregation and leucocyte adhesion may contribute to tumour vascular stasis by promoting endothelial damage and microthrombus formation in the tumour or the feeding vessels. Further studies will be required to determine the mechanism by which NOS inhibition reduces tumour blood flow.

The microvascular effects of substituted arginine NOS inhibitors may be related to the experimental model employed, the underlying vascular reactivity of the host tissues upon which tumours are implanted and competition for substrate within the preparations. For 10–40 μm rabbit tenuissimus muscle venules, Persson *et al.* (1990) reported diameter reductions similar to those we observed, to 82 ± 6% of baseline with 100 μM MeArg; pretreatment with 1 mM L-arginine inhibited the venular diameter reduction. Kubes *et al.* (1991), however, observed no venular diameter reduction in 30 μm cat mesenteric venules superfused with either 100 μM MeArg or 100 μM NoArg. We chose superfusion to avoid systemic cardiovascular effects, however vessel absorption by this route, and thus the intensity of observed effects, may differ from systemic administration. Although we superfused L-arginine in a concentration similar to that reported for normal rat plasma (Albina *et al.*, 1990), it is possible that we did not achieve physiological intracellular levels of L-arginine. This could be a reason for the partial reversal of RBC velocity we observed in control preparation venules.

In addition, inflammatory cells and activated macrophages may, through production of arginase (Albina *et al.*, 1990; Benninghoff *et al.*, 1991), compete for available L-arginine such that higher than physiological concentrations of L-arginine may be required to completely reverse NOS inhibition. L-Arginine can be metabolised either to citrulline in the presence of the calcium-dependent and -independent forms of NOS, producing NO in the process, or to ornithine and urea in the presence of arginase. Levels of arginine in wound fluid from 10-day-old granulating sponge implants are less than 50 μM, as compared with 225 μM in normal rat plasma (Albina *et al.*, 1990); wound fluid ornithine and arginase activity are highest in this model 10 days following sponge implantation as well, suggesting that competition between macrophages and the vascular endothelium for L-arginine may be a factor in granulation tissue models. Further, based on Michaelis–Menten kinetics, concentrations of MeArg higher than 50 μM may be required for maximal inhibition of NOS (Pollock *et al.*, 1991). Optimisation of NOS inhibitor and L-arginine concentration and kinetics will be necessary in order to fully exploit this potential therapy.

We have observed that inhibition of NOS reduces both tumour and normal microvascular perfusion and that subsequent administration of L-arginine can restore normal tissue, but not tumour, perfusion. Tumours may influence their own microvascular perfusion as well as perfusion in surrounding tissues by inducing NOS production. Tumour NOS inhibition followed by normal tissue rescue with administration of L-arginine may provide a novel means of achieving the therapeutic goal of selective tumour hypoxia.

**Abbreviations:** NO, nitric oxide; NOS, nitric oxide synthase; eNOS, endothelial NOS; bNOS, neuronal or brain NOS; iNOS, inducible NOS; MeArg, $N^G$-monomethyl-L-arginine; NoArg, $N^G$-nitro-L-arginine; EBSS, Earle's balanced salt solution; IL, interleukin; TNF, tumour necrosis factor.

**Acknowledgements**
This work was supported by funds from Apex Bioscience, Inc., PO Box 12847, Research Triangle Park, NC 27709-2847, USA, and National Cancer Institute Grant RO1-CA40355. The authors wish to acknowledge the technical assistance of J Edwards and M Mays.

**References**

ALBINA JE, MILLS CD, HENRY WL AND CALDWELL MD. (1990). Temporal expression of different pathways of L-arginine metabolism in healing wounds. *J. Immunol.*, **144**, 3877–3880.

AMBER IJ, HIBBS JR JB, TAINTOR RR AND VAVRIN Z. (1988). The L-arginine dependent effector mechanism is induced in murine adenocarcinoma cells by culture supernatant from cytotoxic activated macrophages. *J. Leukocyte Biol.*, **43**, 187–192.

ANDRADE SP, HART IR AND PIPER PJ. (1992). Inhibitors of nitric oxide synthase selectively reduce flow in tumour-associated neovasculature. *Br. J. Pharmacol.*, **107**, 1092–1095.

BAKER M AND WAYLAND H. (1974). On-line volume flow rate and velocity profile measurement for blood in microvessels. *Microvasc. Res.*, **7**, 131–143.

BENNINGHOFF B, LEHMANN V, ECK H-P AND DROGE W. (1991). Production of citrulline and ornithine by interferon-γ treated macrophages. *Int. Immunol.*, **3**, 413–417.

BROWN JM AND KOONG A. (1991). Therapeutic advantage of hypoxic cells in tumors: a theoretical study. *J. Natl Cancer Inst.*, **83**, 178–185.

BUTTERY LDK, SPRINGALL DR, ANDRADE SP, RIVEROS-MORENO V, HART I, PIPER PJ AND POLAK JM. (1993). Induction of nitric oxide synthase in the neo-vasculature of experimental tumours in mice. *J. Pathol.*, **171**, 311–319.

CHAPLIN DJ. (1989). Hydralazine-induced tumor hypoxia: a potential target for cancer chemotherapy. *J. Natl Cancer Inst.*, **81**, 618–622.

CHAPLIN DJ AND ACKER B. (1987). The effect of hydralazine on the tumor cytotoxicity of the hypoxic cell cytotoxin RSU-1069: evidence for therapeutic gain. *Int. J. Radiat. Oncol. Biol. Phys.*, **16**, 911–917.

CHEN II, PREWITT RL AND DOWELL RF. (1981). Microvascular rarefaction in spontaneously hypertensive rat cremaster muscle. *Am. J. Physiol.*, **241**, H306–H310.

CROWDER MJ AND HAND DJ. (1990). *Analysis of Repeated Measures*. Chapman & Hall: London.

DEWHIRST MW, PRESCOTT DM, CLEGG S, SAMULSKI TV, PAGE RL, THRALL DE, LEOPOLD K, ROSNER G, ACKER JC AND OLESON JR. (1990). The use of hydralazine to manipulate tumour temperatures during hyperthermia. *Int. J. Hyperthermia*, **6**, 971–983.

DEWHIRST MW, SECOMB TW, ONG ET, HSU R, GROSS JF. (1994). Determination of local oxygen consumption rates in tumors. *Cancer Res.*, **54**, 3333–3336.

HILF R, MICHEL I, BELL C, FREEMAN JJ AND BORMAN A. (1965). Biochemical and morphologic properties of a new lactating mammary tumor line in the rat. *Cancer Res.*, **25**, 286–299.

HOGAN JC, LEWIS MJ AND HENDERSON AH. (1988). *In vivo* EDRF activity influences platelet function. *Br. J. Pharmacol.*, **94**, 1020–1022.

IGNARRO LJ, BYRNS RE, BUGA GM AND WOOD KS. (1987). Endothelium-derived relaxing factor from pulmonary artery and vein possesses pharmacologic and chemical properties identical to those of nitric oxide radical. *Circ. Res.*, **61**, 866–879.

KUBES P AND GRANGER DN. (1992). Nitric oxide modulates microvascular permeability. *Am. J. Physiol.*, **262** (*Heart Circ. Physiol.* 31), H611–H615.

KUBES P, SUZUKI M AND GRANGER DN. (1991). Nitric oxide: an endogenous modulator of leukocyte adhesion. *Proc. Natl Acad. Sci. USA*, **88**, 4651–4655.

MONCADA S, PALMER RMJ AND HIGGS EA. (1991). Nitric oxide: physiology, pathophysiology, and pharmacology. *Pharmacol. Rev.*, **43**, 109–142.

PALMER RM, FERRIGE AG AND MONCADA S. (1987). Nitric oxide release acccounts for the biological activity of endothelium-derived relaxing factor. *Nature*, **327**, 524–526.

PAPPENFUSS D, GROSS JF, INTAGLIETTA M AND TREESE FA. (1979). A transparent access chamber for the rat dorsal skin fold. *Microvasc. Res.*, **18**, 311–318.

PERSSON MG, GUSTAFFSON LE, WIKLUND NP, HEDQVIST P AND MONCADA S. (1990). Endogenous nitric oxide as a modulator of rabbit skeletal muscle microcirculation *in vivo*. *Br. J. Pharmacol.*, **96**, 418–424.

PETERSON, H-I. (1991). Modification of tumour blood flow – a review. *Int. J. Radiat. Biol.*, **60**, 201–210.

POLLOCK JS, FORSTERMANN U, MITCHELL JA, WARNER TD, SCHMIDT HHHW, NAKANE M AND MURAD F. (1991). Purification and characterization of particulate endothelium-derived relaxing factor synthase from cultured and native bovine aortic endothelial cells. *Proc. Natl Acad. Sci. USA*, **88**, 10480–10484.

RADOMSKI MW, JENKINS DC, HOLMES L AND MONCADA S. (1991). Human colorectal adenocarcinoma cells: differential nitric oxide synthesis determines their ability to aggregate platelets. *Cancer Res.*, **51**, 6073–6078.

SAS INSTITUTE. (1992). *SAS Technical Report P-229, SAS STAT Software: Changes and Enhancements*, Release 6.07. SAS Institute: Cary, NC.

SCHINI VB, BUSSE R AND VANHOUTTE PM. (1994). Inducible nitric oxide synthase in vascular smooth muscle. *Arzneimittelforschung*, **44**, 432–435.

SECOMB TW, HSU R, DEWHIRST MW, KLITZMAN B AND GROSS JF. (1993). Analysis of oxygen transport to tumor tissue by microvascular networks. *Int. J. Radiat. Oncol. Biol. Phys.*, **25**, 481–489.

SHERMAN PA, LAUBACH VE, REEP BR, WOOD ER. (1993). Purification and cDNA sequence of an inducible nitric oxide synthase from a human tumor cell line. *Biochemistry*, **32**, 11600–11605.

THOMSEN LL, LAWTON FG, KNOWLES RG, BEESLEY JE, RIVEROS-MORENO V AND MONCADA S. (1994). Nitric oxide synthase activity in human gynecological cancer. *Cancer Res.*, **54**, 1352–1354.

TOMPKINS WR, MONTI R AND INTAGLIETTA M. (1974). Velocity measurements of self-tracking correlator. *Rev. Sci. Instrum.*, **45**, 647–649.

WOOD PJ, STRATFORD IJ, ADAMS GE, SZABO C, THIEMERMANN C AND VANE JR. (1993). Modification of energy metabolism and radiation response of a murine tumour by changes in nitric oxide availability. *Biochem. Biophys. Res. Commun.*, **192**, 505–510.

WOOD PJ, SANSOM JM, BUTLER SA, STRATFORD IJ, COLE SM, SZABO C, THIEMERMANN C AND ADAMS GE. (1994). Induction of hypoxia in experimental murine tumors by the nitric oxide synthase inhibitor, $N^G$-nitro-L-arginine. *Cancer Res.*, **54**, 6458–6463.

YAO S-K, OBER JC, KRISHNASWAMI A, FERGUSON JJ, ANDERSON HV, GOLINO P, BUJA LM AND WILLERSON JT. (1992). Endogenous nitric oxide protects against platelet aggregation and cyclic flow variations in stenoses and endothelium-injured arteries. *Circulation*, **86**, 1302–1309.